# Molecular Recycling of Polyethylene Terephthalate (PET) Waste via Ytterbium Triflate-Catalyzed Hydrolysis

**DOI:** 10.1021/acsomega.6c07178

**Published:** 2026-08-11

**Authors:** Pouya Talaei, Phillip E. Savage

**Affiliations:** Robert V. Waltemeyer Department of Chemical Engineering, 8082 The Pennsylvania State University, University Park, Pennsylvania 16802, United States

## Abstract

Returning waste polyethylene terephthalate (PET) to its original monomers (e.g., terephthalic acid (TPA)) constitutes closed-loop molecular recycling. Hydrolysis can accomplish this conversion and ytterbium triflate (Yb­(OTf)_3_) is an effective catalyst. PET hydrolysis with Yb­(OTf)_3_ at 220 °C for 40 min resulted in 92% PET conversion and 66% TPA yield, whereas the uncatalyzed reaction produced just 0.1% TPA yield. We investigated the effects of temperature (160–250 °C), reaction time (6–960 min), catalyst loading (0.9–37 mol % relative to moles of PET repeat units), PET particle size, and PET:water w/w ratio (1:10–1:2) on Yb­(OTf)_3_-catalyzed PET hydrolysis. The PET disappearance kinetics followed a rate law that was pseudo-first-order in PET with a temperature-dependent induction time. The Arrhenius plot showed two regimes. At 205 °C or greater, the activation energy was 54 ± 12 kJ/mol. This regime may reflect the catalytic kinetics for hydrolysis of PET in a softened, swollen, or molten state. The reaction order with respect to Yb­(OTf)_3_ in this regime was 0.77 ± 0.02. For hydrolysis below 205 °C, the activation energy was 165 ± 13 kJ/mol. This regime may reflect the kinetics for PET in a more rigid solid-like state with diffusion of water and/or catalyst within PET being a rate-limiting process. Smaller PET particles reacted more quickly than larger ones in this regime, consistent with diffusion being rate limiting and with the rate being favored by increased PET surface area. Mono-2-hydroxyethyl terephthalate (MHET) was the key reaction intermediate. The PET:water w/w ratio played a crucial role in reaction outcomes, as the TPA yield decreased by about 40% when the ratio was reduced from 1:10 to 1:2.

## Graphical abstract

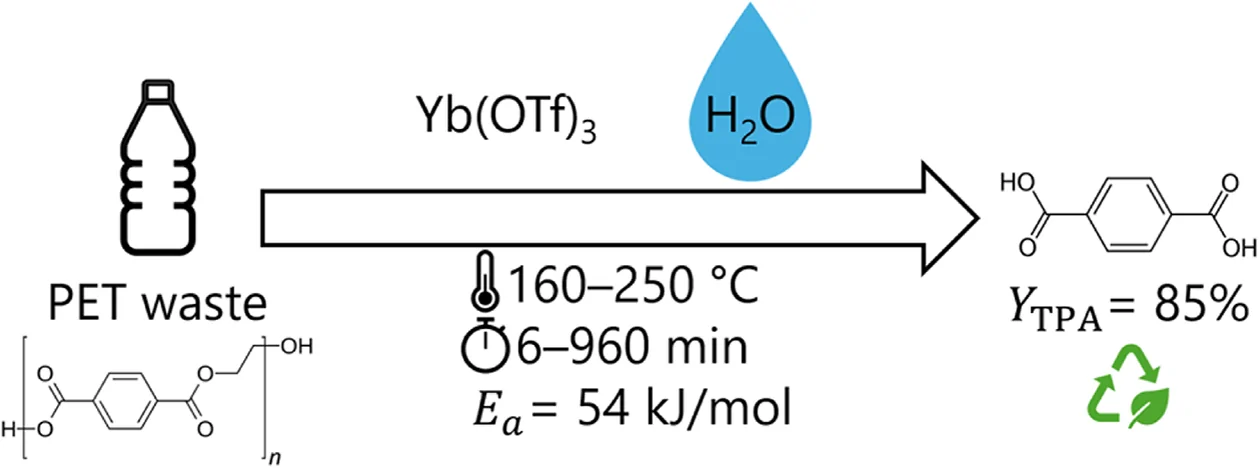

## 1 Introduction

Polyethylene terephthalate (PET), a lightweight polyester with durability, mechanical strength, and transparency, is widely used in packaging, textiles, and construction materials.^1,2^ This widespread use poses significant challenges to the environment. Mechanical recycling, which is the dominant method, supports reuse of postconsumer PET material, but eventually the mechanical properties of the plastic deteriorate, and landfilling will be its ultimate fate.^1,3–5^ Landfilling and incineration both lead to environmental and health concerns.^1,6,7^ Molecular recycling, on the other hand, converts waste PET into its monomers, which can then be used in virgin PET production. This approach eliminates PET waste and landfilling and may enable a circular economy for PET.

One approach for molecular recycling of PET is hydrolysis, which uses water, an inexpensive, nontoxic, and versatile solvent, to depolymerize PET. The ultimate products are terephthalic acid (TPA) and ethylene glycol, the two key monomers used in industrial PET production.^8,9^ PET hydrolysis is slow in neutral water without a catalyst.^8^ Higher temperatures lead to faster rates, but at the expense of increased energy input and in some cases, unwanted product decomposition.^9^ While hydrolysis catalyzed by strong inorganic acids (e.g., sulfuric acid) or strong bases (e.g., NaOH) results in high TPA yields, these approaches pose challenges. Acid hydrolysis generates wastewater containing mineral salts and remaining acids. Alkaline hydrolysis requires an additional acidification step due to the formation of terephthalate salts. Both methods also deal with equipment corrosion, which increases process costs.^1,10,11^ Finding effective and more environmentally friendly catalysts for PET hydrolysis has remained a research goal.

Metal trifluoromethanesulfonates (metal triflates) are a class of water-tolerant Lewis acids that catalyze various organic transformations.^12–17^ They are nontoxic, environmentally friendly, easily recoverable, and reusable without losing their activity. Unlike other Lewis acids (e.g., AlCl_3_), they are stable in water and can be used in catalytic rather than stoichiometric amounts.^18–20^ They can activate the carbonyl group in a reactant even in water.^21^ As Lewis acids, metal triflates coordinate with the carbonyl oxygen in the ester groups of PET and draw electron density away from the carbonyl carbon. This action makes it more electrophilic and facilitates ester bond cleavage through nucleophilic attack by water.^8,22,23^

Rare-earth metal triflates accelerate the hydrolytic depolymerization of poly­(bisphenol A carbonate).^18^ Additionally, complete depolymerization of PET was achieved when metal triflates were used in PET acidolysis.^24^ The only prior work^8^ on the use of metal triflates for PET hydrolysis is from our lab and it explored metal triflates under just one or a few selected reaction conditions. In contrast, the present work provides the first kinetic study of PET hydrolysis catalyzed by a metal triflate, including determination of reaction rate constants, activation energies, and the reaction order with respect to the catalyst. These kinetic parameters are essential for reactor design, process modeling, and scale-up but have not previously been reported for metal triflate-catalyzed PET hydrolysis. Furthermore, this work provides the first assessment of the fate of a metal triflate catalyst during PET hydrolysis.

Although the effects of particle size, water loading, temperature, and reaction time have been investigated for other PET hydrolysis systems, their influence is highly dependent on the catalyst and operating conditions. Prior to this work, these parameters had not been systematically evaluated for metal triflate-catalyzed PET hydrolysis. The resulting data establish quantitative relationships between operating conditions and reaction performance, providing information necessary for process optimization and future scale-up.

Moreover, unlike the previous study, which examined only a single temperature, the present work systematically maps the effects of temperature and reaction time over a broad range of operating conditions. This enables identification of reaction conditions that achieve high TPA yields within relatively short reaction times. Such quantitative information was not available in the previous work and provides valuable guidance for process optimization and future scale-up studies.

In the present work, we focus on ytterbium triflate (Yb­(OTf)_3_), as it was among the metal triflates that exhibited the highest catalytic activity for PET hydrolysis.^8^ It also resulted in a near-neutral solution pH at room temperature,^8^ which could be advantageous at scale because it may reduce the need for highly corrosion-resistant materials of construction and lessen environmental concerns.

## 2 Experimental Section

### 2.1 Materials

Perrier sparkling water bottles (16.9 oz) without caps and labels were the source of PET in this study. The bottles were emptied, washed, and dried. The body of each bottle, 0.5 mm thick, was manually cut into small chips with average dimensions of 6 ± 2 mm × 8 ± 2 mm. These chips were used in all experiments reported herein unless indicated otherwise. Other experiments used smaller PET particles that were obtained via grinding the larger chips using a Wiley Mill.^25^ The small particles were subjected to sequential sieving using meshes with pore sizes of 2, 1, 0.5, 0.125, and 0.063 mm to determine their size distribution. The screens retained 0.0, 3.0, 61, 35, and 0.9 wt % of the particles on each sieve, respectively. Therefore, most of the particles (61 wt %) fall within the 0.5–1 mm range, corresponding to an average surface-to-volume (S/V) ratio of approximately 4.5. Most of the remaining particles (35 wt %) are even smaller and in the 0.125–0.5 mm range, which has an average S/V ratio of about 15. Figure S4a and Figure S5a provide images of the initial PET particles and chips, respectively. Previous work^5^ indicated the PET melting point was 250 °C, and the crystallinity was 38% for the body of the bottle. The number-average molecular weight (*M*_n_) of bottle-grade PET is 24000–36000 g/mol.^26^ More details on the PET characterization are available elsewhere.^5^

Ytterbium triflate were purchased from Sigma-Aldrich. Deionized (DI) water was supplied through an in-house purification system. Figure S1 shows that Yb­(OTf)_3_ seemed fully dissolved in water as the catalyst solution was clear. Dimethyl sulfoxide (DMSO) was obtained from Fisher Scientific. All chemicals were used as received. Swagelok stainless steel reactors were assembled from a port connector and two caps with nominal diameters of 1/2 in, resulting in an internal volume of 4 mL.

### 2.2 Experimental Procedure

For the base-case experiments, each reactor was loaded with m_PET_ = 207 mg of PET (1.08 mmol PET repeat units, calculated using a repeat-unit molar mass of 192.17 g/mol), 0.055 mmol of Yb­(OTf)_3_, and 2.07 mL of DI water. The catalyst amount corresponds to a 5.1 mol % loading with respect to PET repeat units. The PET to water mass ratio was 1:10. In some experiments, the amounts of catalyst or water were varied to determine their effects on the reaction outcomes. The loaded reactors were sealed, shaken a few times by hand, and placed in an isothermal Techne fluidized sand bath at the desired temperature for the desired reaction time. It takes about 2.5 min for the temperature in the reactor to reach the set point temperature of the sand bath.^9^ The reactors were removed from the sand bath after the reaction time had elapsed and immediately immersed in room-temperature water to quench the reaction. Each reaction condition was explored in three independent experiments to provide mean values and uncertainties (standard deviations) for product yields.

Ten mL of DI water, in smaller aliquots, was added to each cool reactor after opening and subsequently withdrawn via pipet with the postreaction material. The product slurry passed through a Whatman grade 1 cellulose filter paper, held by a reusable Cole-Parmer filter holder with a diameter of 25 mm. The aqueous filtrate was collected in a Petri dish. The Petri dishes were then dried either in a 45 °C oven or at ambient temperature in a fume hood with flowing air. For one set of runs, the aqueous filtrate and the fresh catalyst solution were analyzed by inductively coupled plasma atomic emission spectrometry (ICP-AES; Thermo iCAP 7400) to determine their ytterbium (Yb) concentrations.

The filter paper with the solids it retained and all other filtration supplies that had been used were placed in an 80 °C oven overnight for drying. These dried water-insoluble solids were collected from all surfaces using DMSO to dissolve TPA and byproducts. The DMSO aliquots were collected in a centrifuge tube. The tube contents were well mixed using a Fisher Scientific digital vortex mixer to facilitate product dissolution and then passed through Agilent nylon syringe filters (25 mm diameter and a 0.45 μm pore size). Material that was retained on the filter and other equipment would include unconverted PET, oligomers, and byproducts that were not soluble in either water or DMSO. We refer to this material as undissolved solids (US) and take it to represent the unreacted PET. The PET yield and conversion (X) were calculated from eqs 1 and 2.

1 $$Y_{\mathrm{PET}}\;(\%)=\frac{m_{\mathrm{US}}}{m_{\mathrm{PET}}}\times 100$$

2 $$X=1-Y_{\mathrm{PET}}$$

The DMSO solution with TPA and any byproducts was diluted 25-fold prior to analysis. Quantification of TPA and mono-2-hydroxyethyl terephthalate (MHET) was via a 1 μL injection into a Shimadzu high-performance liquid chromatograph (HPLC) with a Waters reversed-phase symmetry C_18_ column (5 μm particle size, 150 mm × 4.6 mm) at 40 °C and an SPD-M20A photodiode array detector operating at 240 nm. The mobile phase was HPLC-grade acetonitrile at a flow rate of 0.1 mL/min and a 0.1 vol % of formic acid aqueous solution at 0.3 mL/min.

Standard solutions of TPA and MHET in DMSO at concentrations of 0.5, 1, 2, 5, 8, and 10 mM and 0.0125, 0.025, 0.05, 0.1, 0.2, 0.5, 0.8, 1, 2, and 5 mM, respectively, were analyzed to obtain the calibration curves. The TPA molar yield was calculated from eq 3 as the mass of TPA formed (from HPLC analysis) divided by the maximum mass of TPA possible based on the stoichiometry of the reaction and assuming the feed is 100% PET with TPA as the sole aromatic monomer.

3 $$Y_{\mathrm{TPA}}\;(\%)=\frac{m_{\mathrm{TPA}}}{0.86m_{\mathrm{PET}}}\times 100$$

The factor of 0.86 is the ratio of molecular weights of TPA and the PET repeat unit. An equation of the same form was used for the molar yield of MHET, but with a factor of 1.09.

## 3 Results and Discussion

### 3.1 Control Experiments

These experimental methods for TPA recovery were assessed through a control experiment. Reactors were loaded with TPA, ytterbium triflate, ethylene glycol, and water and held at 220 °C for 40 min. The product recovery and analysis protocol outlined above gave a TPA recovery of 95.0 ± 0.8%, indicating nearly quantitative recovery of TPA.

### 3.2 Temporal Variation of Product Yields

Figure 1 presents the temporal variations of the TPA and PET yields from PET hydrolysis at different temperatures, with a fixed 5.1 mol % Yb­(OTf)_3_ loading. This catalyst loading corresponded to a solution pH of ∼6 at room temperature, and due to the temperature-dependent density of liquid water, catalyst concentrations of 0.021–0.024 M at the reaction temperatures examined. The dashed lines on the plots for TPA are included to guide the eye of the reader. The dashed curves on the plot for PET are calculated from a kinetics model discussed in the next section.

**Figure 1:**
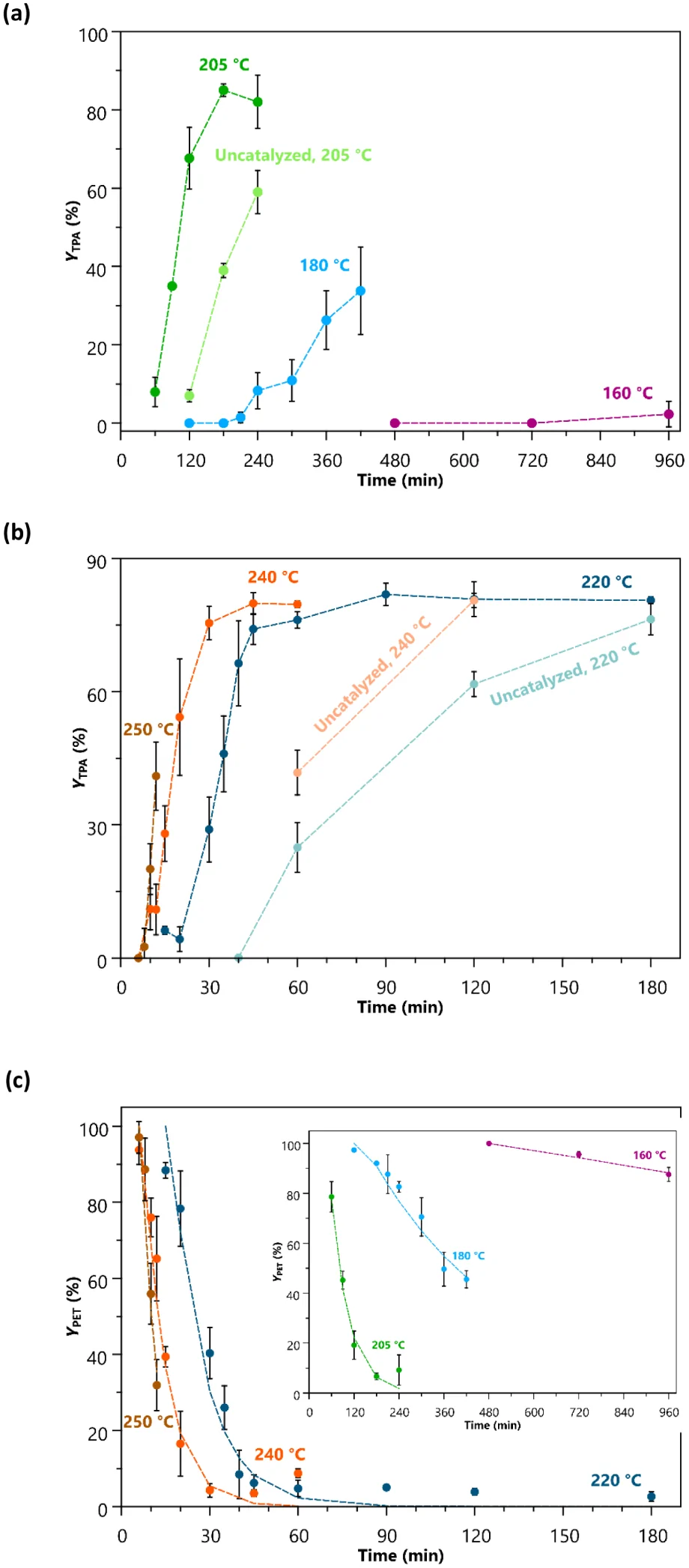
Temporal variation of product yields from PET hydrolysis (1:10 w/w PET:water loading, 5.1 mol % Yb­(OTf)_3_ loading except for uncatalyzed runs) (a) TPA at 160, 180, and 205 °C; (b) TPA at 220, 240, and 250 °C; (c) PET.

Uncatalyzed reactions were also completed to highlight the effect of the catalyst. For uncatalyzed reactions at 205 °C, TPA yields were only 7.0 ± 1.5% and 39 ± 2% after 120 and 180 min, respectively. These low values are consistent with literature.^5^ In contrast, Yb­(OTf)_3_ produced TPA yields of 68 ± 8% and 85 ± 2% after 120 and 180 min, respectively. The yield of TPA from PET hydrolysis at 220 °C for 40 and 60 min was only 0.1 ± 0.1% and 25 ± 6%, respectively, without a catalyst, but it was 66 ± 10% and 76 ± 2%, respectively, when using Yb­(OTf)_3_. Similarly, at 240 °C, the uncatalyzed reaction produced a TPA yield of 42 ± 5% after 60 min, while 76 ± 4% yield was achieved after only 30 min in the presence of Yb­(OTf)_3_.

Catalyzed hydrolysis at 160 °C proceeded very slowly. Even at 960 min, the TPA yield was just 2.3 ± 3.2% and the PET conversion was 12 ± 3%. At 180 °C, there was no measurable TPA yield for the first 180 min, but the yield then increased nearly linearly with time, eventually reaching 34 ± 11% at 420 min. The PET conversion was 54 ± 4%, indicating that the selectivity to TPA was less than 100% at these conditions.

At 205, 220, and 240 °C, TPA yields exceeded 65% and PET conversions exceeded 80% after 120, 40, and 20 min, respectively, when using Yb­(OTf)_3_. For catalyzed hydrolysis at 205–240 °C, the TPA yields seem to reach a plateau around 80% at long reaction times, but the PET conversions are 95% or greater. These observations are consistent with an equilibrium existing among some of the reaction products, as advanced by McNeeley and Liu^27^ and by Pereira et al.^28^ For example, reversible hydrolysis/esterification reactions can establish an equilibrium involving mono-2-hydroxyethyl terephthalate (MHET) and water with TPA and ethylene glycol.

HPLC analyses showed that mono-2-hydroxyethyl terephthalate (MHET) was a frequent byproduct from PET hydrolysis. Figure S2 shows a representative HPLC chromatogram. Bis­(2-hydroxyethyl) terephthalate (BHET) was observed in some runs, but always with much smaller peaks that represented negligible amounts. Figure 2 presents the temporal variation of the MHET yield from hydrolysis at 220 °C for both uncatalyzed and Yb­(OTf)_3_-catalyzed reactions.

**Figure 2:**
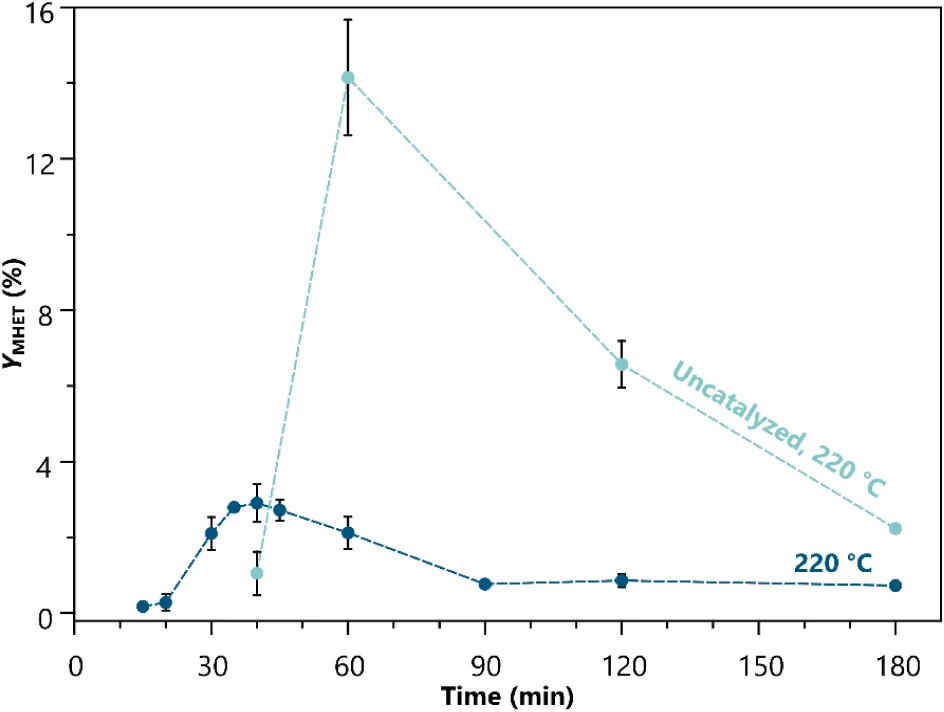
Temporal variation of MHET yields from PET hydrolysis at 220 °C (1:10 w/w PET:water loading, 5.1 mol % Yb­(OTf)_3_ loading except for uncatalyzed runs).

MHET behaves as a reaction intermediate, being first produced and then converted to subsequent products. MHET yields from uncatalyzed hydrolysis were higher than those from catalyzed reactions at the same reaction time. This trend is expected because uncatalyzed reactions proceed more slowly. Yb­(OTf)_3_ accelerates the cleavage of ester bonds in PET, and it should also promote the cleavage of the ester bond in MHET, thereby reducing the amount of this byproduct and increasing the TPA yield in the catalyzed runs. Figure S3 shows that MHET yields for other hydrolysis temperatures display the same trends as in Figure 2.

### 3.3 Kinetics of PET Disappearance

The data for PET yields at different times and temperatures in the previous section permits analysis of the kinetics of PET conversion during Yb­(OTf)_3_-catalyzed PET hydrolysis. Combining the design equation for the constant-volume batch reactors used in the experiments with a power-law rate equation (presumed for convenience) for PET disappearance leads to

4 $$r_{\mathrm{PET}}=-\frac{dC_{\mathrm{PET}}}{dt}=kC_{\mathrm{PET}}^{\alpha }C_{C}^{\beta }C_{W}^{\gamma }$$

where *C*_PET_, *C*_C_, and *C*_W_ are the PET, catalyst, and water concentrations, respectively, and α, *β,*and γ are the respective reaction orders. If we take the rate to be pseudo-first-order in PET, take the water concentration to be constant, write *C*_PET_ in terms of conversion (*X*), and define *k*′ = *k* $C_{C}^{\beta }C_{W}^{\gamma }$ , the resulting differential equation can be integrated and rearranged to give eq 5 as the relationship between conversion and batch holding time for experiments at a fixed catalyst concentration.

5 $$\ln\left(1/\left(1-X\right)\right)=k^{\prime }t$$

Plotting ln­(1/(1 – *X*)) versus time for a given reaction temperature should provide a line with slope equal to the pseudo-first-order rate constant (*k*′). Figure 3 shows the results and Table 1 lists the resulting rate constants.

**Figure 3:**
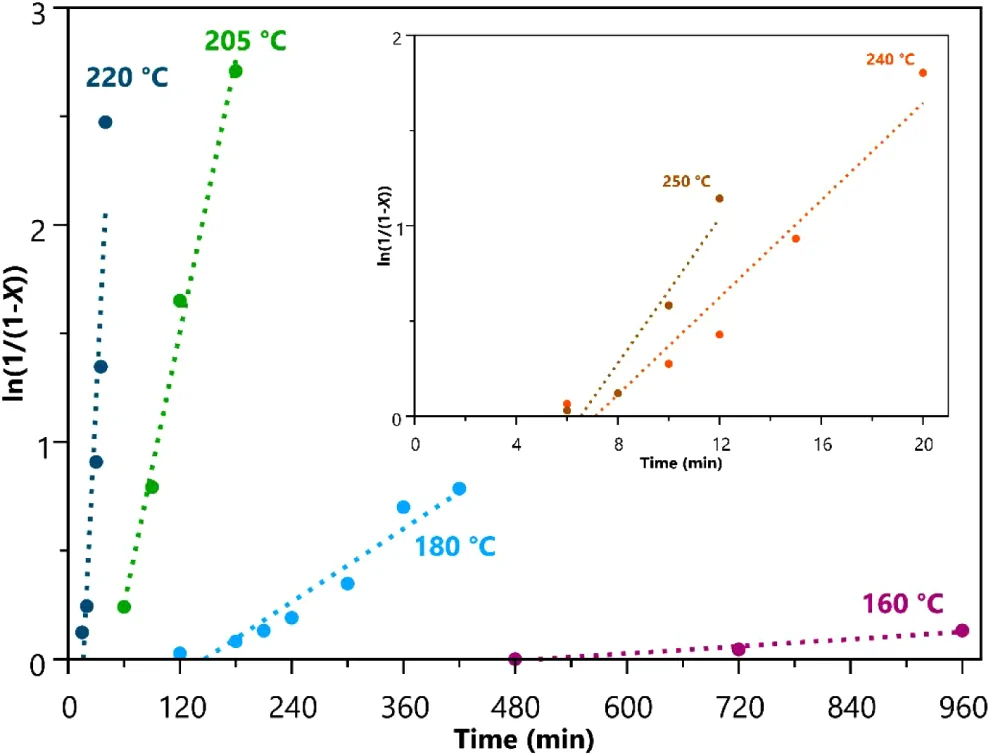
PET disappearance for Yb­(OTf)_3_-catalyzed hydrolysis follows pseudo-first-order kinetics with an induction time (1:10 w/w PET:water loading, 5.1 mol % Yb­(OTf)_3_ loading).

**Table 1:** Pseudo-First-Order Rate Constants and Induction Times for PET Hydrolysis^a^

*T* (°C)	*k*′ (*min* ^–1^)	*t_i_* (min)
160	2.75(±0.51) × 10^–4^	506
180	2.81(±0.33) × 10^–3^	146
205	2.09(±0.14) × 10^–2^	48.0
220	8.69(±1.74) × 10^–2^	16.3
240	1.28(±0.19) × 10^–1^	7.11
250	1.90(±0.38) × 10^–1^	6.53

a (1:10 w/w PET:water loading, 5.1 mol % Yb­(OTf)_3_ loading).

The fitted lines did not pass through the origin, as one would expect from eq 5. We interpret this nonzero intercept as indicating the presence of an induction period for this catalyzed hydrolysis reaction. Table 1 lists the observed induction times, and they decrease from 506 min for hydrolysis at 160 °C to 6.5 min for hydrolysis at 250 °C. We used the rate constants and induction times in Table 1 to calculate the curves for PET disappearance in Figure 1c.

The literature contains prior reports of induction times for PET hydrolysis.^29^ These initial periods of time during which there is no measurable PET conversion may correspond to the time required for penetration of enough water and catalyst within the PET to initiate cleavage of ester bonds, although other factors, such as catalyst speciation (discussed later), surface activation, and the progressive formation of accessible reaction sites, may also contribute. The induction times decreasing as temperature increases could be due to the elevated temperatures more rapidly softening the polymer and better allowing transport of solvent and catalyst into and within the PET. Although the PET melting point is around 250 °C in vacuum, it reportedly softens at temperatures as low as 200 °C in hot, compressed water.^30^ Based on these in situ thermal measurements, we expect the PET chips to be in a softened or molten state at temperatures above 200 °C and in a more rigid, solid-like state at lower temperatures.

Figure 4 shows the Arrhenius plot for Yb­(OTf)_3_-catalyzed hydrolysis of PET from this work along with literature data for uncatalyzed hydrolysis in neutral water.^5^ The catalytic kinetics are consistent with the existence of two kinetic regimes. One corresponds to lower temperatures where PET may be in a more solid-like state and the other at higher temperatures where PET may be in a softened state. The apparent activation energy in the low-temperature regime was 165 ± 13 kJ/mol. Diffusion of water and catalyst into and within the PET is likely the controlling process in this region. Yb­(OTf)_3_ is much larger than water molecules, so its diffusion within the polymer matrix is slower and thus more impactful on the observed rate. The activation energy for diffusion of different organic molecules within PET at 120–180 °C has been reported to be around 116–194 kJ/mol.^31^ The apparent activation energy we measure falls within this range and supports the possibility of the hydrolysis rate being limited by catalyst diffusion in this region.

**Figure 4:**
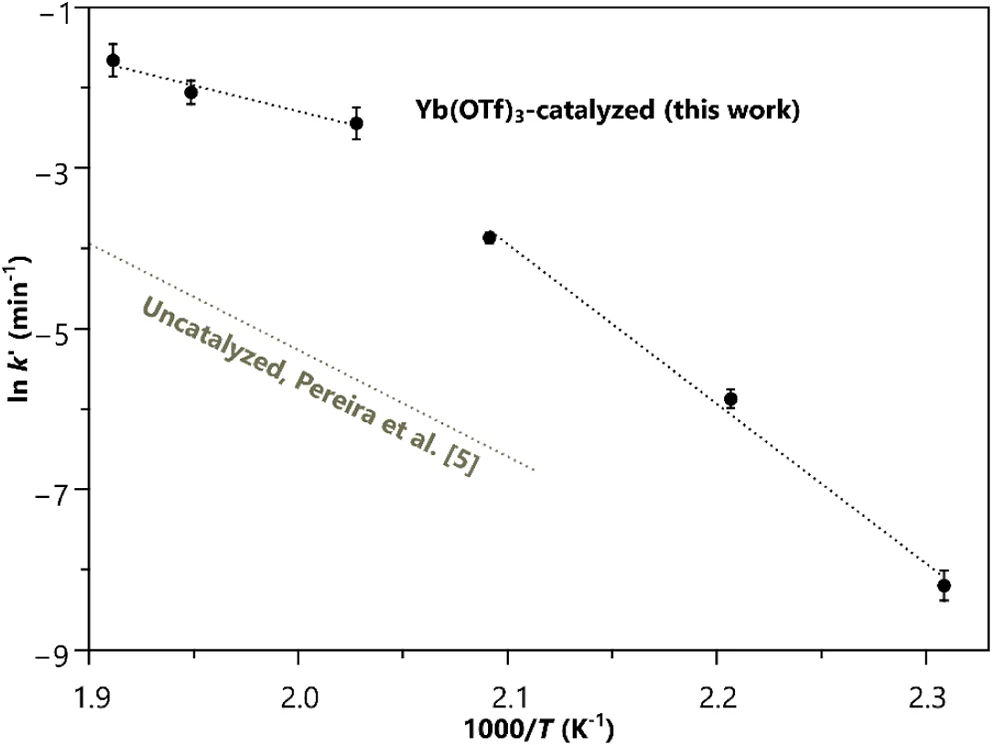
Arrhenius plot for PET disappearance during hydrolysis (1:10 w/w PET:water loading, 5.1 mol % Yb­(OTf)_3_ loading).

The activation energy is 54 ± 12 kJ/mol in the higher-temperature regime. As expected for a catalytic reaction, this value is lower than the apparent activation energy 110 ± 13 kJ/mol for uncatalyzed PET hydrolysis^5^ at T ≥ 200 °C. Yb­(OTf)_3_ effectively lowers the energy barrier for the cleavage of ester bonds in PET.

Images of the unreacted PET particles recovered after reaction provide qualitative evidence consistent with the proposed two-regime reaction. Figure S4 shows the PET particles recovered from reactions conducted at 180 °C for 240 min (27% PET conversion) largely retained their shape and size. PET particles recovered after reaction at 240 °C for 6 min (6% PET conversion), however, were far fewer and substantially larger, consistent with PET undergoing softening and agglomeration during this brief time at 240 °C.

In addition, Figure S5b,c shows that the recovered PET chips from reactions under the proposed solid-state, diffusion-controlled regime largely retained their original shape and glossy appearance. In contrast, Figure S5e–g shows that the recovered PET material from reactions within the proposed molten/swollen-state regime appeared markedly different from the original PET chips loaded into the reactors, even though the extents of PET conversion are about the same for all images.

We next wanted to determine the effect of the catalyst concentration (at the reaction temperature) on the rate of PET hydrolysis. The catalyst concentration was assumed to remain constant throughout the reaction. We did experiments at 220 °C for 30 min with one catalyst loading higher than that used in the base-case experiments and one loading that was lower. Under these conditions, the PET conversion with the base-case catalyst concentration (0.022 M) was ∼60%, which would allow the effect of changing the catalyst concentration to be evaluated. The corresponding pseudo-first-order rate constants were calculated using 13.7 min as the reaction time (30 min holding time minus 16.3 min induction time) and then plotted against catalyst concentration on a log–log plot (Figure 5). The slope of a line through the data provides the catalyst reaction order (*β*) at 220 °C as 0.77 ± 0.02.

**Figure 5:**
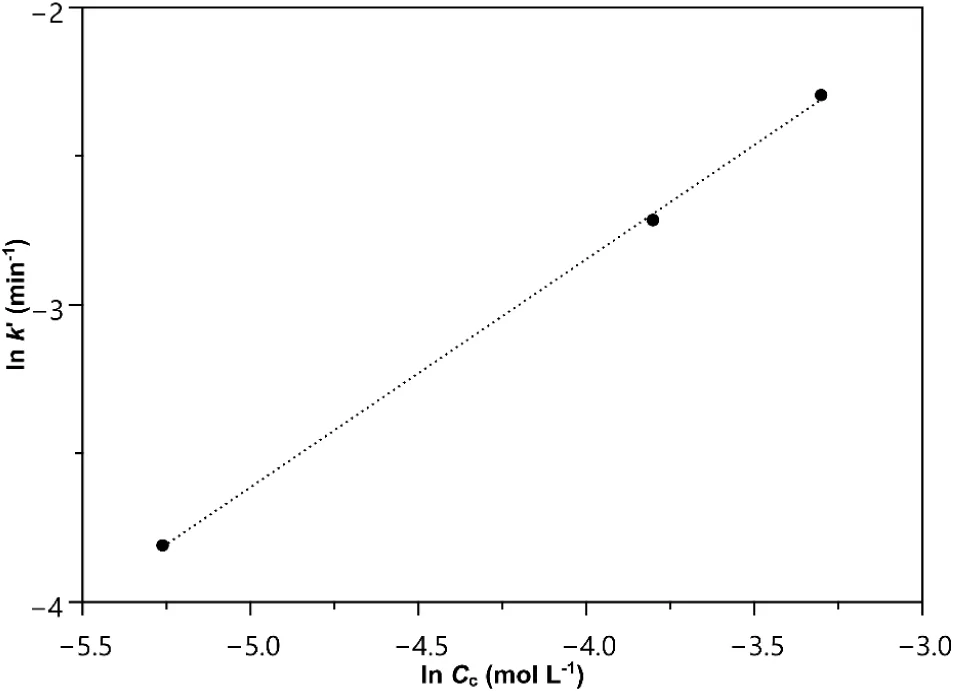
Effect of Yb­(OTf)_3_ concentration on pseudo-first-order rate constant (220 °C, 30 min, 1:10 w/w PET:water).

Reactions catalyzed by metal triflates are often first order in catalyst,^20,32^ but fractional reaction orders can arise when the metal also forms less active or inactive species in water. In these instances, the catalyst speciation would lead to the reaction order being less than unity, as in the present study. We discuss here the possible aqueous speciation of Yb­(OTf)_3_ based on previous studies of water-compatible lanthanide Lewis acids.^33–38^ Upon dissolution in water, Yb­(OTf)_3_ is expected to dissociate to form predominantly hydrated ytterbium­(III) ions.^33–35^ These hydrated species are believed to be the starting point for Lewis acid catalysis in aqueous media.^33,35^ The coordinated water molecules are not static but undergo rapid exchange with molecules in the solution.^33^ Consequently, oxygen-containing substrates such as the ester carbonyl groups of PET may temporarily replace one or more coordinated water molecules, allowing direct coordination of the carbonyl oxygen to the ytterbium center and thereby increasing the electrophilicity of the carbonyl carbon toward nucleophilic attack by water.^33,36^

At the same time, the hydrated ytterbium species may undergo partial hydrolysis to form aqua–hydroxo complexes.^33,37^ This reaction also releases protons, introducing the possibility that Brønsted acid catalysis contributes alongside Lewis acid activation.^37^ Therefore, the observed catalytic activity may arise from one or more hydrated or partially hydrolyzed ytterbium species rather than from a single well-defined molecular catalyst.^33,37^ Furthermore, as PET hydrolysis proceeds, reaction products with oxygen-containing functional groups, particularly terephthalic acid and its carboxylate forms, may transiently coordinate to ytterbium because lanthanide ions exhibit a strong affinity for carboxylate ligands.^38^ Such interactions could alter the distribution of ytterbium species in solution and potentially influence catalyst availability and activity.^38^

These dynamic speciation equilibria may contribute to the experimentally observed fractional reaction order with respect to the catalyst. If the relative abundances of hydrated, hydrolyzed, substrate-bound, and product-bound ytterbium species vary with catalyst concentration, solution composition, or reaction progress, the concentration of catalytically active species would not necessarily increase proportionally with the total catalyst concentration. Such behavior could lead to an apparent fractional reaction order even if the intrinsic catalytic step associated with the active species is first order in catalyst. Additional work with direct characterization of catalyst speciation under hydrothermal conditions is needed to distinguish among these possibilities.

We also examined the influence of catalyst concentration (at the reaction temperature) on the yield of TPA at 220 °C for 45 min. Figure S6 shows that the TPA yield increased from 59 ± 8% to 84 ± 1% as the catalyst concentration increased from ∼0.01 to ∼0.2 M. As noted in Section 3.2, uncatalyzed PET hydrolysis at 220 °C for a longer reaction time (60 min) produced a TPA yield of only 25 ± 6%, so even at the lowest catalyst concentration examined, Yb­(OTf)_3_ clearly accelerates PET hydrolysis.

### 3.4 Yb Retention in the Postreaction Aqueous Phase

In previous work, Pereira et al.^8^ tested whether the aqueous solution with Yb­(OTf)_3_ catalyst that was recovered after a reaction could be used again to catalyze a subsequent run. They added fresh water as needed to maintain the same total fluid volume in each run. They report the TPA yield decreased from ∼80% in the first two runs to ∼18% after six cycles (each at 200 °C, 120 min, 1:10 w/w PET:water loading).^8^

To explore this topic further, we had the postreaction aqueous phase from one of our experiments (220 °C, 60 min, 1:10 w/w PET:water loading, and 5.1 mol % Yb­(OTf)_3_ loading) analyzed for its Yb content. Under these conditions, the PET conversion and TPA yield were ∼95% and ∼77%, respectively and 87 ± 8% of the Yb loaded into the reactor remained in the aqueous phase after reaction. This result shows that the concentration of Yb in the recycled aqueous phase might decrease with each reuse cycle. This decrease could then contribute to the decline in TPA yield observed by Pereira et al.^8^ We also attempted to quantify residual ytterbium in the recovered TPA-containing solids by ICP-AES. Regrettably, the material was resistant to complete digestion under all the available analytical procedures, so no measurement could be made.

### 3.5 Effect of Particle Size

The size of the plastic particle can affect the rate of PET hydrolysis if the reaction is limited by the amount of plastic surface area available or the rate of diffusion within the particle. For these scenarios, reducing the PET particle size should enhance the hydrolysis rate. We conducted experiments using smaller PET particles compared to the base-case PET chips to interrogate this possibility. The surface-to-volume (S/V) ratio for the base-case chips was 4.6 mm^–1^, whereas the average S/V ratio for the particles was 8.4 mm^–1^. These values were calculated assuming that the base-case chips are rectangular prisms and the small particles are spheres.

For PET hydrolysis at 220 °C for 30 min, Figure S7 shows that the TPA yields and PET conversions of the base-case PET chips were statistically indistinguishable from those obtained using PET particles that were smaller. An additional experiment at 240 °C for 6 min gave a PET conversion using base-case PET chips of 6.2 ± 3.8% with no detectable TPA formation. When smaller PET particles were used, PET conversion (6.2 ± 8.8%) and TPA yield (0.4 ±0.5%) showed no change. This lack of a particle size effect for hydrolysis at 220 and 240 °C is consistent with prior work for uncatalyzed PET hydrolysis at 200 °C for 120 min.^5^

We conducted additional experiments using the smaller PET particles at 180 °C for 240 min. At this lower temperature, PET is expected to remain predominantly in a rigid, solid-like state, where transport limitations within the polymer and the available surface area may have a greater influence on hydrolysis performance. As shown in Figure 6, the TPA yield using smaller particles was statistically different and higher than that observed with the base-case chips. The same experiment was performed at 180 °C for 120 min. This resulted in a TPA yield of ∼5%, whereas no TPA formation was observed even after 180 min when base-case PET chips were used at this temperature. These outcomes are consistent with previous reports that observed enhanced PET hydrolysis upon reducing particle size at temperatures between 50 and 150 °C.^39–43^ The presence of a particle size effect for PET hydrolysis at 180 °C and its absence at 220 and 240 °C is likely due to the PET being in a softened, more accessible form at higher temperatures, rather than existing in a more dense, solid-like state. The softer or expanded PET would be more amenable to water and catalyst molecules diffusing within the polymer matrix, thereby reducing the influence of particle size on hydrolysis performance.

**Figure 6:**
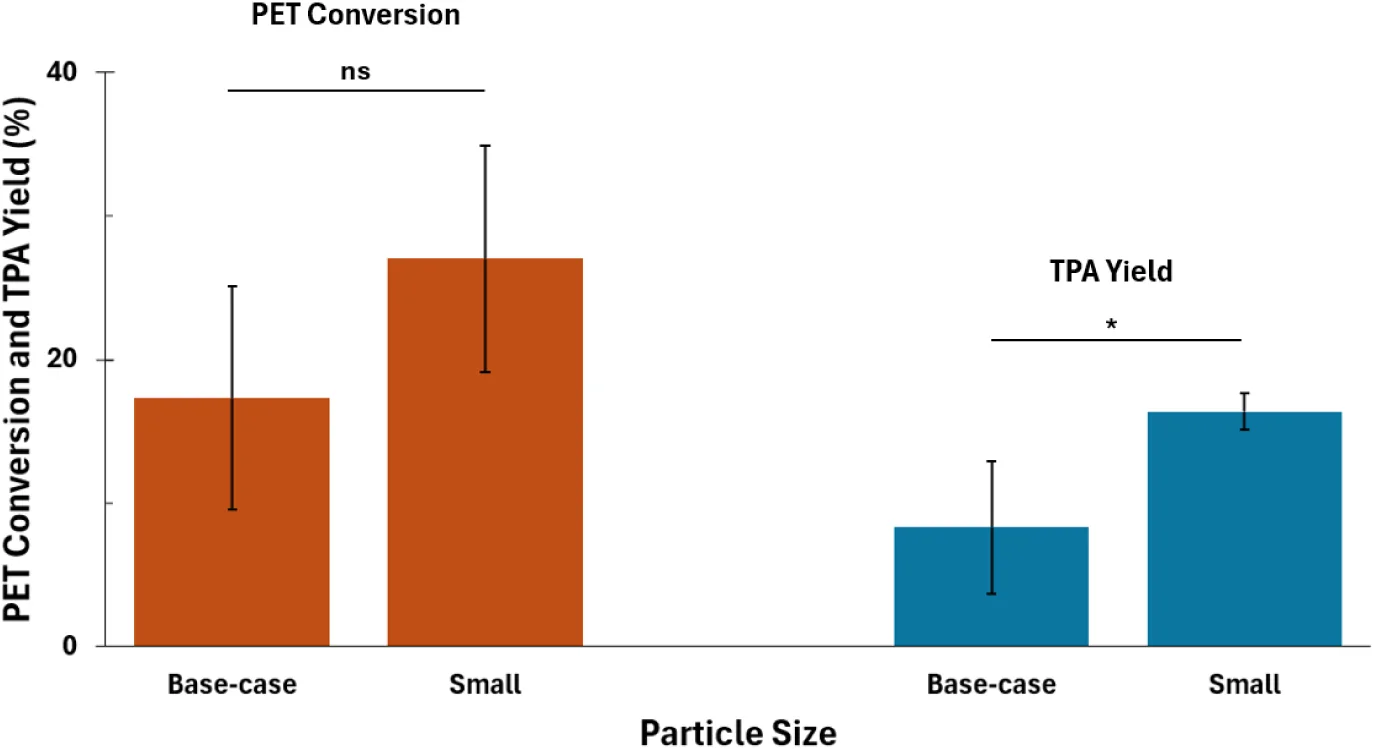
Effect of particle size on PET conversion and TPA yield from PET hydrolysis (180 °C, 240 min, 1:10 w/w PET:water loading, 5.1 mol % Yb­(OTf)_3_ loading). ns denotes a difference that is not statistically significant (*p* > 0.05). * denotes a statistically significant difference (*p* < 0.05).

### 3.6 Effect of PET:Water Loading

The amount of water used in PET hydrolysis is important for a process at scale due to environmental considerations arising from water treatment and its effect on capital and operating expenses. Experimental studies often use PET:water w/w ratios around 1:10, but more PET-rich systems would be preferred at scale. We examined the effect of this process variable on PET conversion and TPA yield for Yb­(OTf)_3_-catalyzed PET hydrolysis at 220 °C for 40 min. For each experiment, the masses of PET and catalyst were kept constant, and different masses of water were loaded into the reactor to achieve the desired w/w ratio with PET. Approximately 99%, 98%, and 90% of the water was in the liquid phase under the reaction conditions for the PET:water w/w ratios explored (1:10, 1:6, and 1:2, respectively).^44^

Figure 7 shows that PET conversion and TPA yield decline as the system becomes more PET rich. This behavior occurred in uncatalyzed hydrolysis as well.^9^ The uncertainties are large enough, however, that the difference in yield between the 1:10 w/w ratio and 1:6 is not statistically significant. Using a 1:2 w/w loading does lead to a statistically significant decrease in yield (21 ± 11% vs 66 ± 10%). The reduction in PET conversion and TPA yield is not caused by stoichiometric limitations, as a PET:water w/w ratio of 1:0.189 is stoichiometrically sufficient for complete depolymerization. There must be other factors contributing to the observed behavior. As the PET:water loading increased in the experiments, the amount of water added decreased. Perhaps this reduction in liquid-phase volume led to poorer contact between the aqueous-phase catalyst and the solid polymer or increased transport resistances due to the increasingly concentrated reaction medium. Detailed multiphase transport modeling of this system could be helpful in identifying the physical and chemical causes of the observed reduction in yield and conversion in concentrated systems.

**Figure 7:**
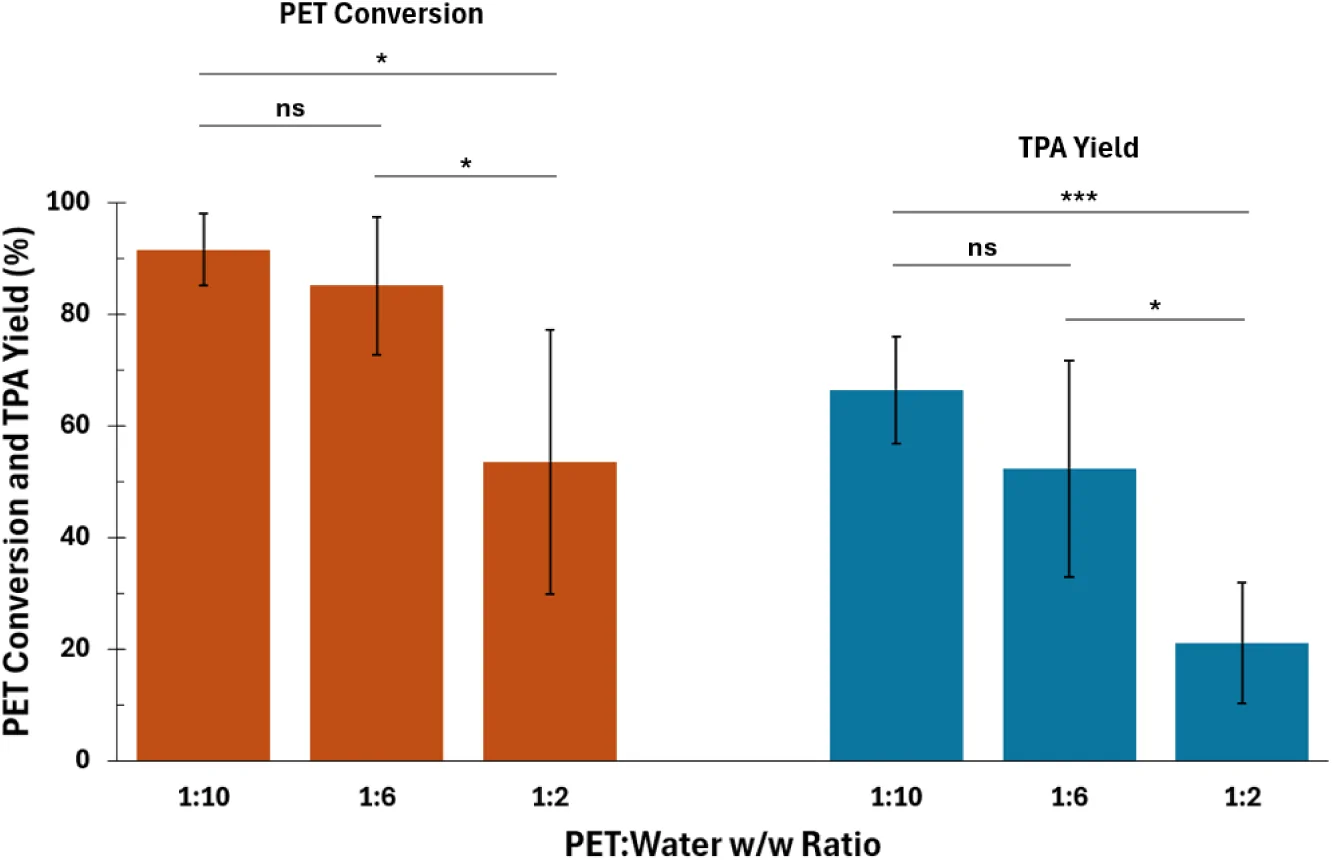
Effect of PET:water w/w ratio on PET conversion and TPA yield from PET hydrolysis (220 °C, 40 min, 5.1 mol % Yb­(OTf)_3_ loading). ns denotes a difference that is not statistically significant (*p* > 0.05). * and *** denote statistically significant differences at *p* < 0.05 and *p* < 0.001, respectively.

## 4 Considerations for Triflate-Catalyzed Hydrolysis of PET at Scale

As with any process that uses a catalyst that is soluble in the reaction medium, catalyst recovery is an important consideration at scale. Likewise, given the large, superstoichiometric volume of water needed for PET hydrolysis, recovering, treating, and recycling the water is an important consideration. Water and catalyst recovery are complicated by ethylene glycol and other water-soluble byproducts also being present in the postreaction aqueous phase. Distillation could be used to produce a water recycle stream from the overhead, but with high energy costs. Distillation would also produce a bottoms stream of ethylene glycol with dissolved catalyst.^27^ Recovery of catalyst alone would require an additional separation (e.g., vacuum evaporation of ethylene glycol or its derivatization into a molecule immiscible with water followed by water addition to recover the catalyst).

Because ytterbium triflate is a relatively expensive catalyst, its recovery and reuse are expected to be important for the economic viability of the process. Consequently, future process development should evaluate not only catalyst activity but also catalyst stability and recovery. These factors should be incorporated into techno-economic and life cycle assessments to identify economically and environmentally viable process configurations and operating conditions.

## 5 Conclusions

Yb­(OTf)_3_ exhibited catalytic activity for PET hydrolysis, achieving TPA yields of up to ∼85%. TPA yields of ∼70% were obtained at 205, 220, and 240 °C in only 120, 40, and 20 min, respectively, substantially outperforming uncatalyzed hydrolysis under comparable conditions.

Reactions at 160 and 180 °C were significantly slower. At 160 °C, the TPA yield reached only ∼2% after 960 min. The slow rates in this temperature range are likely due to PET existing in a state with reduced accessibility by water and catalyst to ester bonds within the polymer structure. Consistent with this observation, an induction time was observed at each temperature when modeling the reaction kinetics using a pseudo-first-order approach. Further, hydrolysis of PET particles with larger S/V ratio proceeded more quickly in this regime, supporting the importance of available surface area for the hydrolysis reaction.

The Arrhenius plot revealed two kinetic regimes, with apparent activation energies of 165 ± 13 kJ/mol at lower temperatures and 54 ± 12 kJ/mol at higher temperatures. As expected, the Yb­(OTf)_3_ catalyst lowers the apparent activation energy for hydrolysis in the higher-temperature regime, where uncatalyzed PET hydrolysis studies are typically conducted. The reaction order with respect to ytterbium triflate in this regime was 0.77 ± 0.02. The value being less than unity may reflect changes in catalyst speciation under reaction conditions. Characterization of the postreaction medium could provide further insight into this behavior.

Finally, reducing the PET:water w/w ratio from 1:10 to 1:2 resulted in a substantial decrease in both PET conversion and TPA yield. Additional work is needed to identify the PET:water w/w ratio that best optimizes the conceptual process at scale.

## Supplementary Material



## Notes

It includes images of some of the materials used, a representative chromatogram, yields for MHET, TPA yields with different catalyst concentrations, and PET particle sizes (PDF)
