# Supplementary material for: Molecular Recycling of Polyethylene Terephthalate (PET) Waste via Ytterbium Triflate-Catalyzed Hydrolysis
Source: ACS Omega. 2026 Aug 11;11(33):50561–9. doi: 10.1021/acsomega.6c07178 (PMC13520748; doi:10.1021/acsomega.6c07178)
Supplement: Supplementary file 1 [file ao6c07178_si_001.pdf]

**Supplementary Information**

# Molecular Recycling of Polyethylene Terephthalate (PET) Waste via Ytterbium Triflate-Catalyzed Hydrolysis

**Pouya Talaei, Phillip E. Savage***

*Robert V. Waltemeyer Department of Chemical Engineering, The Pennsylvania State University, University Park, PA 16802, USA*

* corresponding author

**Number of pages: 5**

**Number of figures: 7**

**Number of tables: 0**

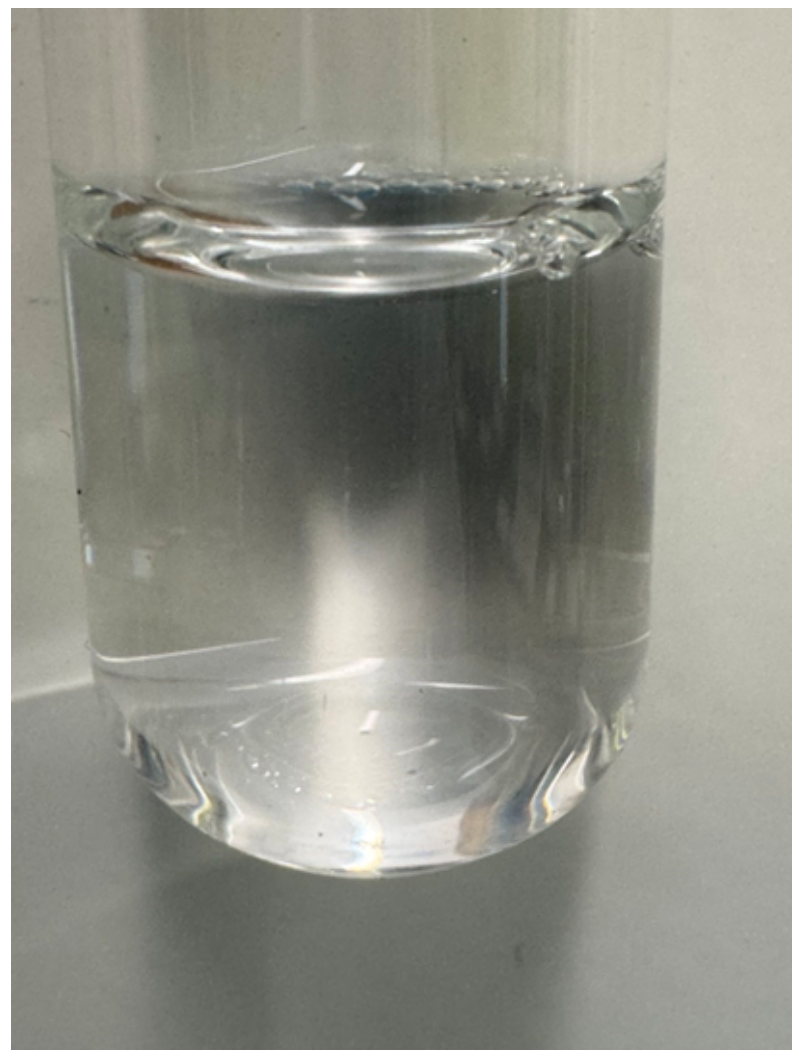

**Figure S1.** Image of aqueous solution of $Yb(OTf)_3$ (0.026 M) at room temperature.

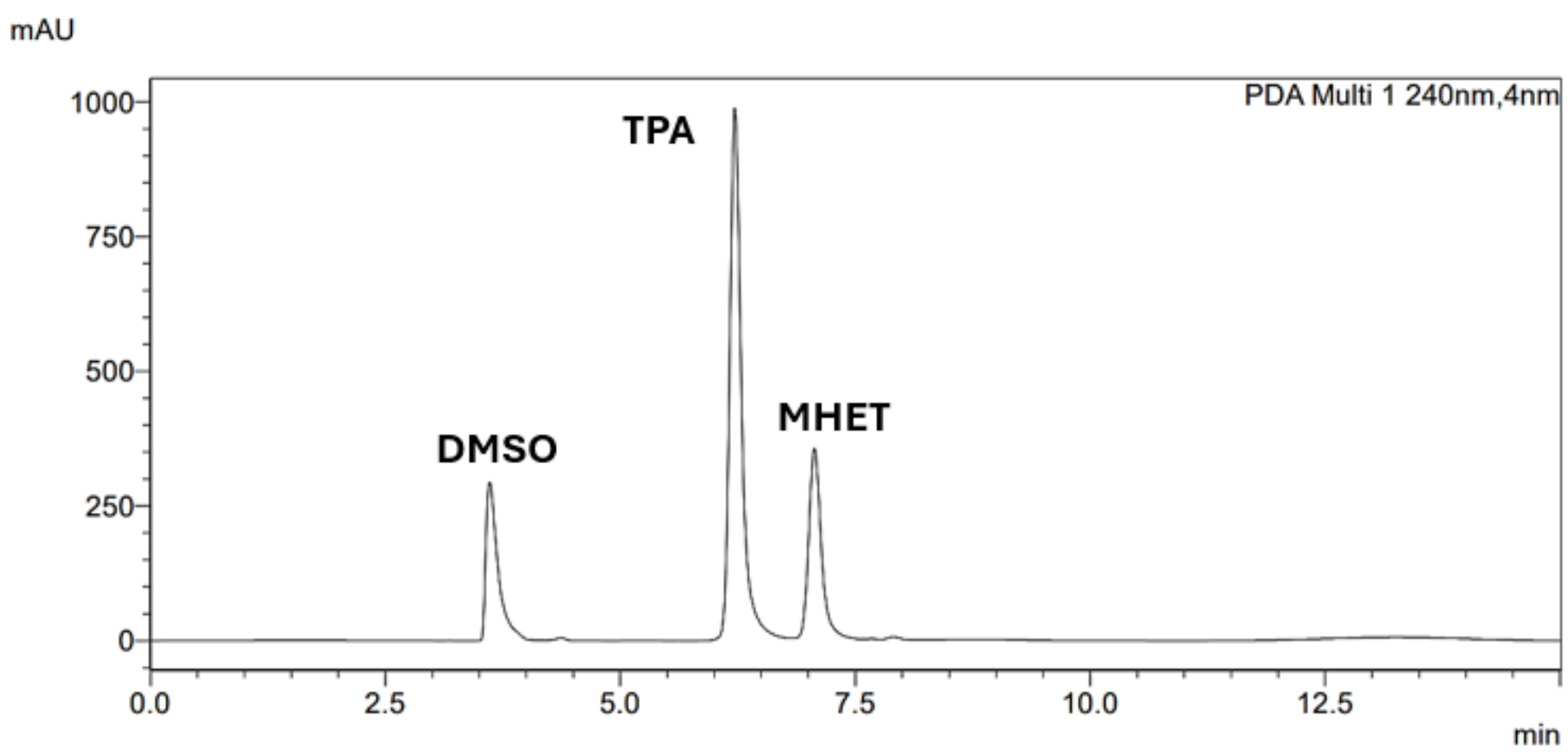


**Figure S2.** High-performance liquid chromatogram for DMSO-soluble products from uncatalyzed PET hydrolysis (220 °C, 60 min, 1:10 w/w PET:Water).

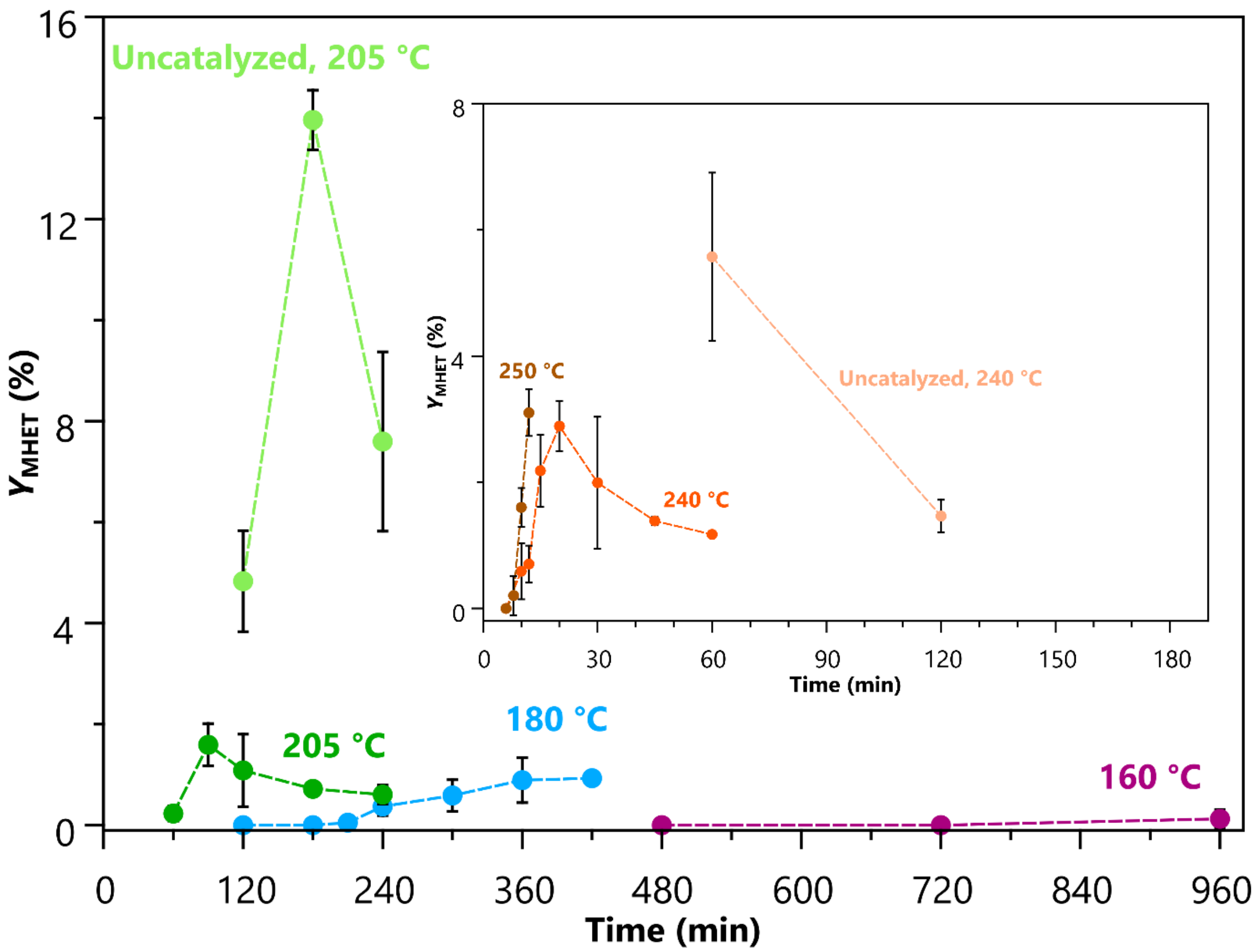


**Figure S3.** Temporal variation of MHET yield from PET hydrolysis (1:10 w/w PET:water loading, 5.1 mol% $Yb(OTf)_3$ loading except for uncatalyzed runs).

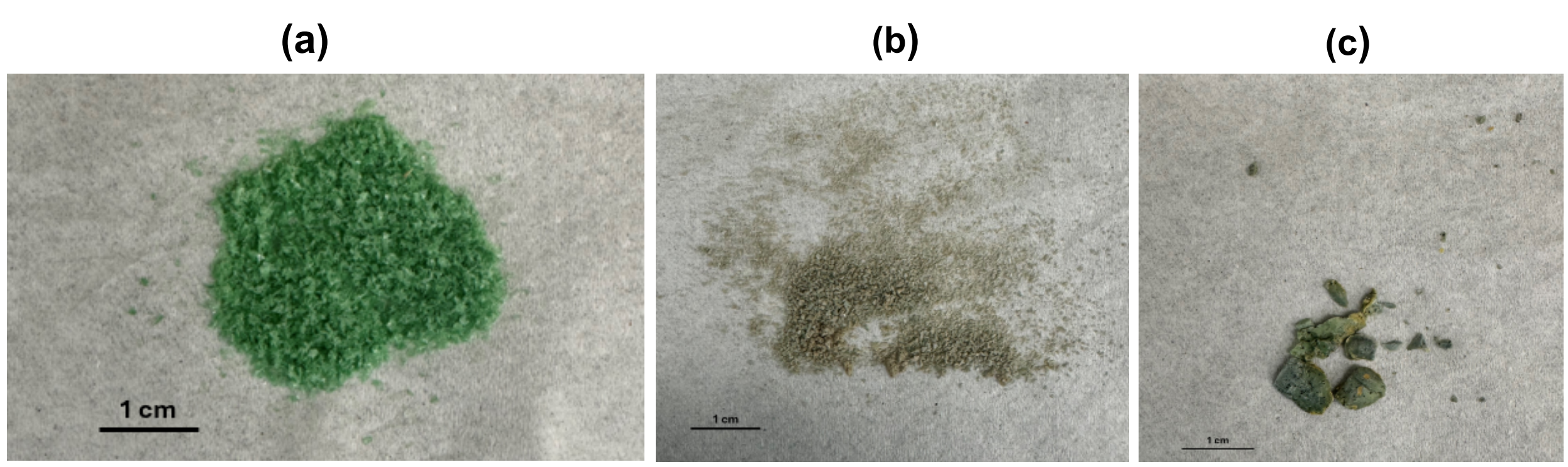


**Figure S4.** PET particles (a) fresh, as used in the experiments; (b) after reaction at 180 °C for 240 min (27% PET conversion) and (c) after reaction at 240 °C for 6 min (6% PET conversion). (1:10 w/w PET:water loading, 5.1 mol% $Yb(OTf)_3$ loading).

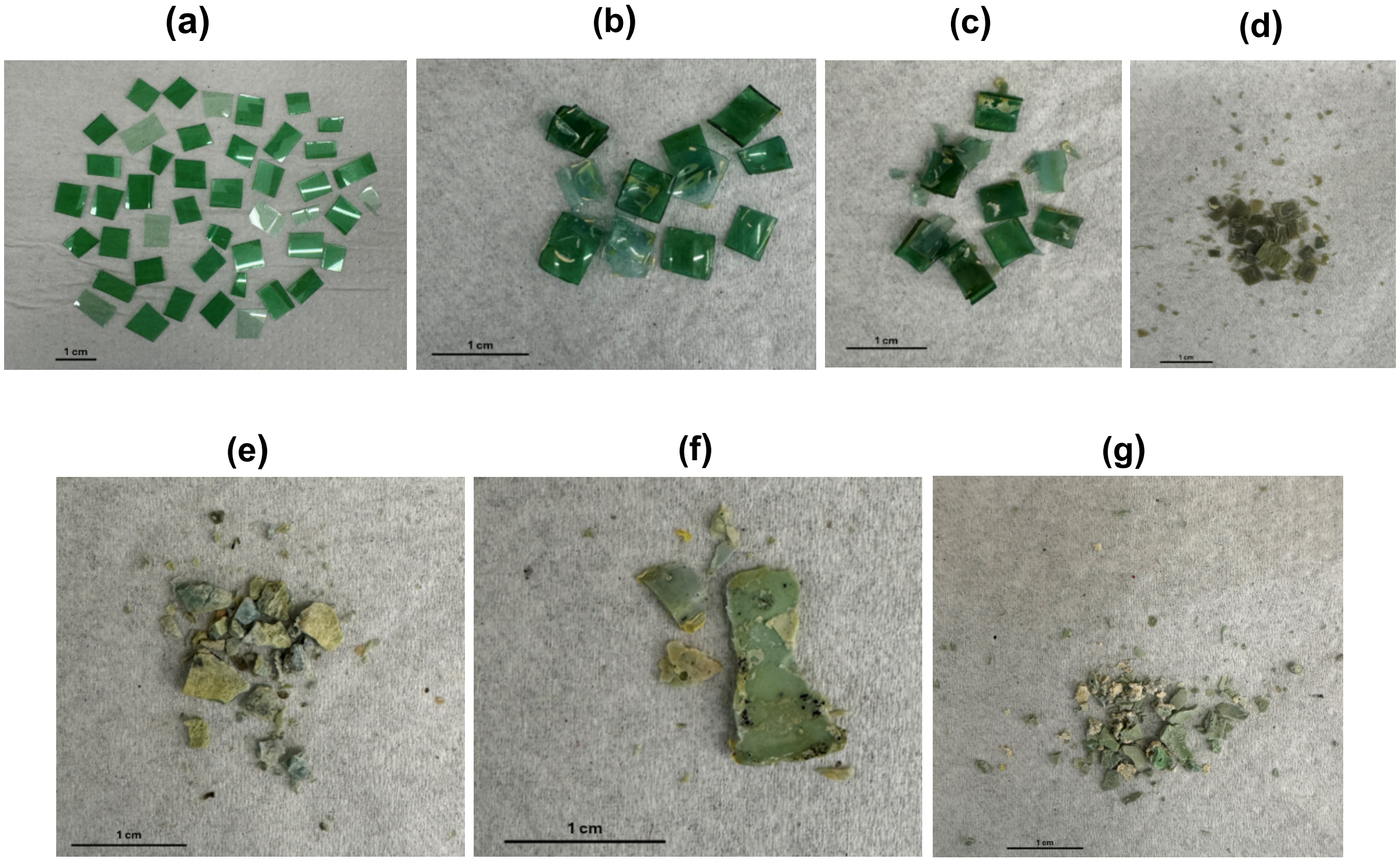


**Figure S5.** PET chips (a) fresh, as used in the experiments; and after reaction at: (b) 160 °C for 360 min (0% PET conversion); (c) 180 °C for 120 min (3% PET conversion); (d) 205 °C for 60 min (17% PET conversion); (e) 220 °C for 15 min (9% PET conversion); (f) 240 °C for 6 min (0% PET conversion); and (g) 250 °C for 6 min (0% PET conversion). (1:10 w/w PET:water loading and a 5.1 mol% $Yb(OTf)_3$ loading.)

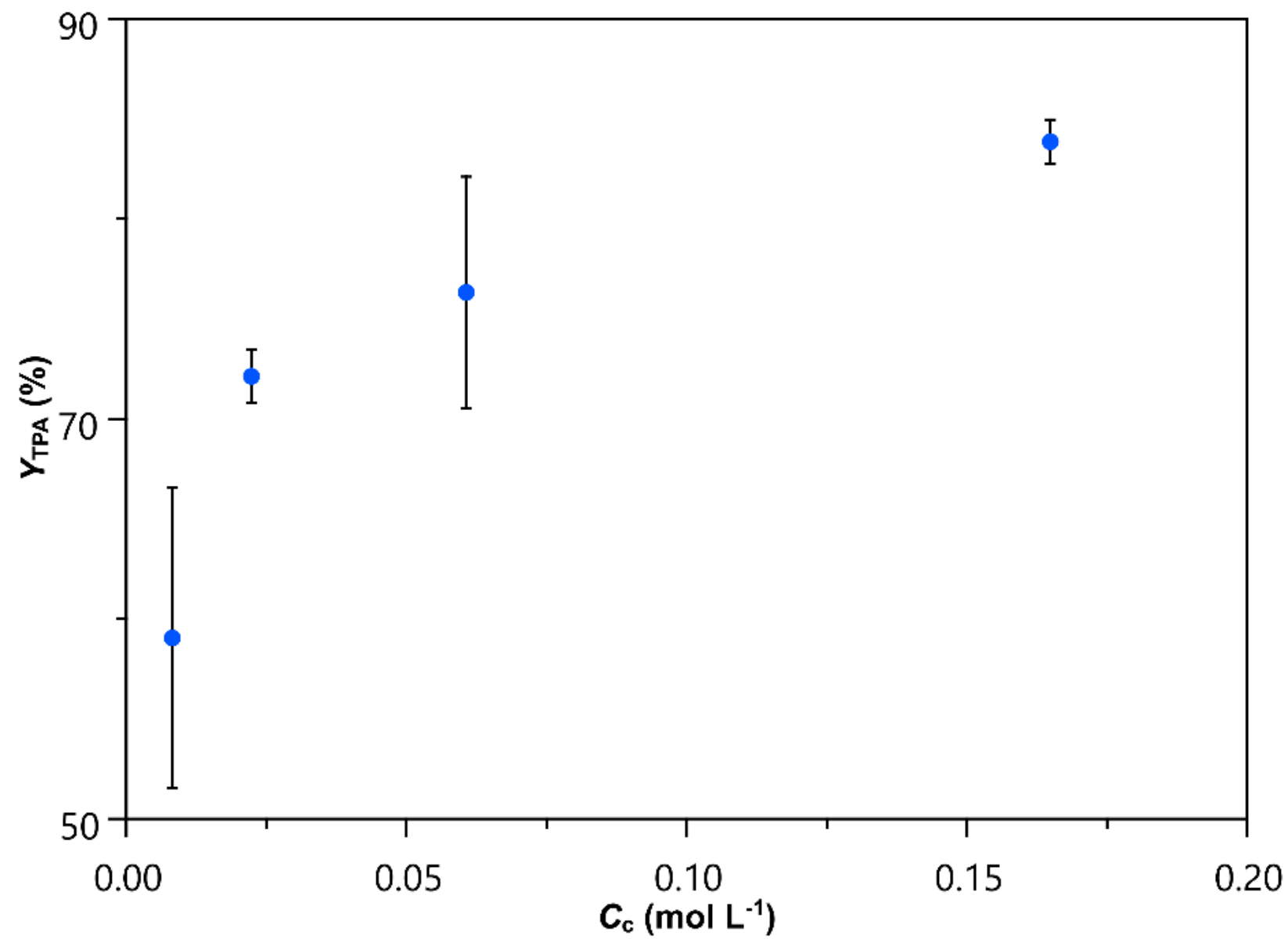


**Figure S6.** Effect of $Yb(OTf)_3$ concentration on TPA yield (220 °C, 45 min, 1:10 w/w PET:Water).

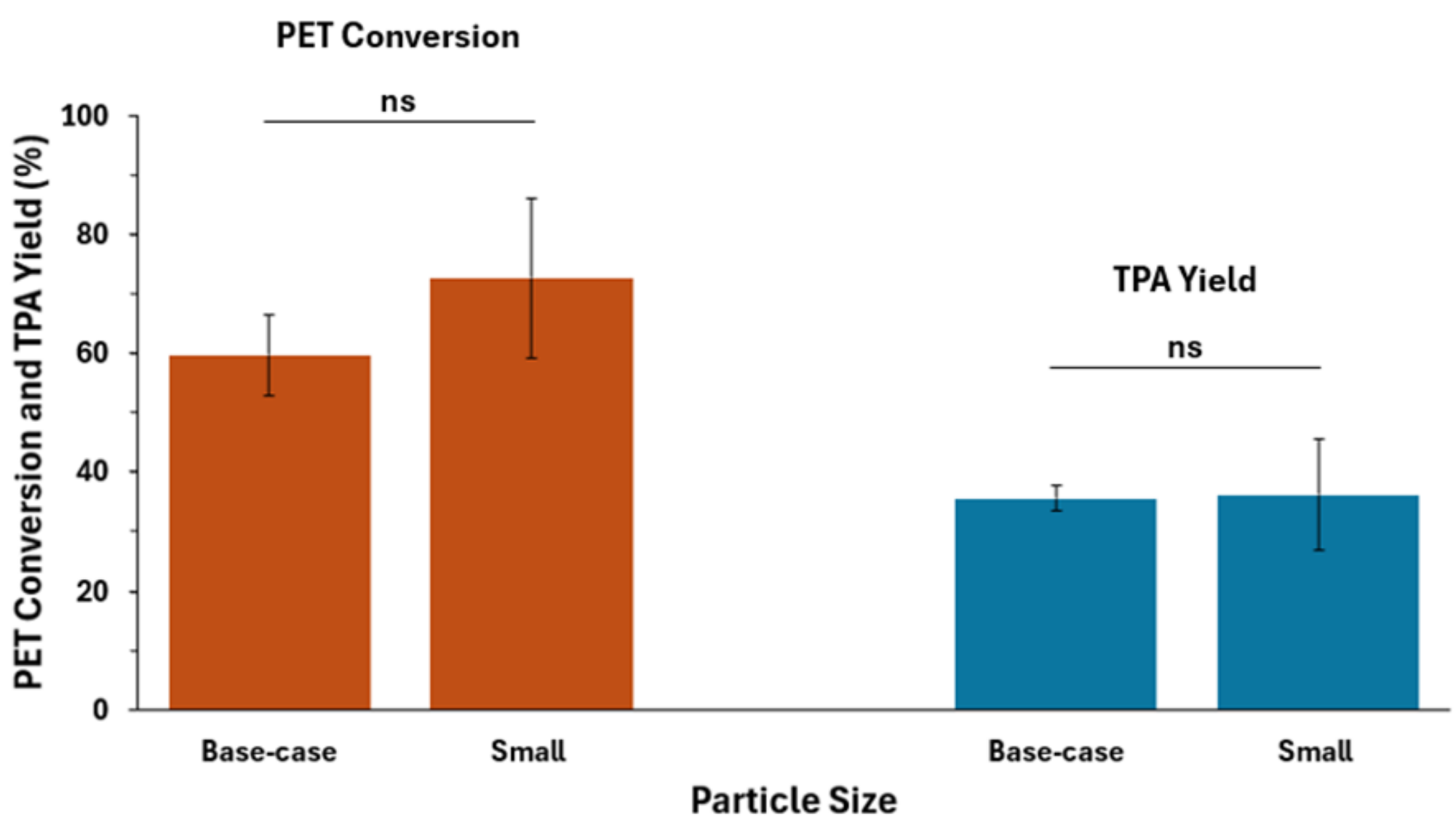


**Figure S7.** Effect of particle size on PET conversion and TPA yield from PET hydrolysis (220 °C, 30 min, 1:10 w/w PET:water loading, 5.1 mol% $Yb(OTf)_3$ loading). ns denotes a difference that is not statistically significant ($p > 0.05$).
